# An AD‐associated variant in *TTC3* alters the actin cytoskeleton organization and synaptic function in iPSC‐derived forebrain neurons

**DOI:** 10.1002/alz.092304

**Published:** 2025-01-03

**Authors:** Holly N. Cukier, Carolina L. Duarte, Lauren Coombs, Caleb Avery Lambert, Derek Van Booven, Brooke A. DeRosa, Margaret A Pericak‐Vance, Anthony J. Griswold, Derek M. Dykxhoorn

**Affiliations:** ^1^ John P. Hussman Institute for Human Genomics, University of Miami Miller School of Medicine, Miami, FL USA; ^2^ University of Miami, Miami, FL USA; ^3^ John P. Hussman Institute for Human Genomics, Miami, FL USA; ^4^ John P. Hussman Institute for Human Genomics, Miller School of Medicine, Miami, FL USA; ^5^ 1501 NW 10th Avenue, Miami, FL USA; ^6^ Dr. John T. Macdonald Foundation Department of Human Genetics, University of Miami Miller School of Medicine, Miami, FL USA; ^7^ John P. Hussman Institute for Human Genomics, University of Miami Miller School of Medicine, Miami, FL, USA, Miami, FL USA

## Abstract

**Background:**

We identified the missense variant Ser1038Cys (rs377155188) in the *tetratricopeptide repeat domain 3* (*TTC3*) gene that segregate in a non‐Hispanic white late onset Alzheimer disease (LOAD) family. This variant is predicted to be deleterious and extremely rare (MAF<0.01%). Expression of *TTC3* is decreased in the brains of LOAD patients and negatively correlated with AD neuropathology.

**Methods:**

CRISPR/Cas9 genome editing was used to introduce the TTC3 Ser1038Cys variant into a control iPSC line. induced pluripotent stem cells (iPSCs) were developed that were homozygous for the TTC3 variant and validated for pluripotency. To understand the mechanism(s) by which this TTC3 variant may contribute to LOAD risk, this isogenic pair of edited and unedited iPSCs was differentiated into forebrain neurons and assessed for the cellular, molecular, and transcriptional consequences of this variant, including neuronal morphology, synaptic functionality, and vesicular trafficking.

**Results:**

Neuronal progenitor cells (NPCs) derived from the isogenic iPSC lines showed that TTC3 variant‐bearing cells had elevated migration rates compared to their wild type counterparts as assessed by wound healing assays. Forebrain neurons bearing the Ser1038Cys variant showed elongated neurites and higher numbers of branchpoints compared to the wild type neurons phenocopying the effect of TTC3 loss of function analysis (siRNA‐mediated silencing of TTC3 in mouse neurons). This phenotype could be reversed using small molecule modulators of actin polymerization. Immunoblot analysis showed alterations in the expression of pre‐ and post‐synaptic markers in the TTC3 Ser1038Cys neurons compared to control neurons. RNA‐seq analysis of day 70 neurons identified 978 differentially expressed protein‐coding genes (FDR<0.05), including known AD genes (*BACE1*, *A2M*, *INPP5F*, *UNC5C*) and genes in AD GWAS loci (*ADAMTS1*, *MAF, NCK2*). Pathway analysis identified differential expression in PI3K‐Akt signaling pathway, as well as genes involved in axon guidance, the GABAergic synapse, and the Wnt signaling pathways.

**Conclusions:**

The results of these studies suggest that the TTC3 p.Ser1038Cys variant causes a loss of function leading to alterations in neurite outgrowth, branching, and synapse formation, phenotypes consistent with alterations in actin stability. These studies in iPSC‐derived neurons implicate alterations in actin as potential mechanisms by which TTC3 may contribute to LOAD risk.

